# Cytokines and the Skin Barrier

**DOI:** 10.3390/ijms14046720

**Published:** 2013-03-26

**Authors:** Kai H. Hänel, Christian Cornelissen, Bernhard Lüscher, Jens Malte Baron

**Affiliations:** 1 Institute of Biochemistry and Molecular Biology, Medical School, RWTH Aachen University, Pauwelsstrasse 30, 52074 Aachen, Germany; E-Mails: khaenel@ukaachen.de (K.H.H.); christian.cornelissen1@rwth-aachen.de (C.C.); 2 Department of Dermatology and Allergology, Medical School, RWTH Aachen University, Pauwelsstrasse 30, 52074 Aachen, Germany

**Keywords:** skin barrier, cytokine, cornification, atopic dermatitis, psoriasis, interleukin, filaggrin, keratin, involucrin, keratinocytes

## Abstract

The skin is the largest organ of the human body and builds a barrier to protect us from the harmful environment and also from unregulated loss of water. Keratinocytes form the skin barrier by undergoing a highly complex differentiation process that involves changing their morphology and structural integrity, a process referred to as cornification. Alterations in the epidermal cornification process affect the formation of the skin barrier. Typically, this results in a disturbed barrier, which allows the entry of substances into the skin that are immunologically reactive. This contributes to and promotes inflammatory processes in the skin but also affects other organs. In many common skin diseases, including atopic dermatitis and psoriasis, a defect in the formation of the skin barrier is observed. In these diseases the cytokine composition within the skin is different compared to normal human skin. This is the result of resident skin cells that produce cytokines, but also because additional immune cells are recruited. Many of the cytokines found in defective skin are able to influence various processes of differentiation and cornification. Here we summarize the current knowledge on cytokines and their functions in healthy skin and their contributions to inflammatory skin diseases.

## 1. Introduction

The skin is the largest organ in the human body and its major function is the protection from harmful environmental influences and to prevent dehydration. It forms the first barrier against physical, biological and chemical stress. To maintain this function keratinocytes undergo a differentiation process from proliferating basal cells, which arise from epidermal stem cells, to spinous, granular, and transitional cells, and finally culminates in the generation of corneocytes. These are highly differentiated, nucleus-free cells that form an insoluble and rigid structure referred to as the cornified envelope (CE). The correct formation of the CE is essential for the barrier function of the skin [1]. This differentiation program relies on well-regulated cell communication processes. Keratinocytes and other skin resident cells produce cytokines that are responsible for the control of cellular communication. Cytokine signaling can result in multiple consequences for the barrier function of the skin. For example cytokines influence keratinocyte proliferation and differentiation, at least in part by modulating the gene expression program in these cells. One consequence is the expressional control of other cytokines resulting in a complex network of signaling molecules that affect the physiology of keratinocytes and the quality of the skin barrier. Deregulated cytokine expression can thus contribute to dysfunctions of the epidermal barrier as it is observed in many diseases, including atopic dermatitis (AD) and psoriasis.

AD is an inflammatory skin disease with pruritic, eczematous erythematous plaques [2] affecting 10%–20% of children and 1%–3% of adults in industrialized countries [3]. Psoriasis is characterized by dry, bright red plaques with thick, non-adherent, silver-white scales [2]. The prevalence of psoriasis in children and in adults is estimated to be up to 0.7% and 3% in the EU and US, respectively [4]. These are just two examples of inflammatory skin diseases that involve defects in skin barrier formation, with both being extensively studied regarding their molecular pathology. Also other skin diseases like ichthyosis [5] or urticaria [6] exhibit defects in barrier formation leading to an aggravation of disease symptoms.

The inflammatory milieu of these diseases is well characterized. However, the function of the involved cytokines and the contributions of their downstream signaling pathways and cross-talk between these pathways to disease progression are only partially understood. Here we summarize the current state of research concerning cytokines involved in disturbing the formation of the physical skin barrier.

## 2. Key Features of the Epidermal Barrier

**Figure 1:**
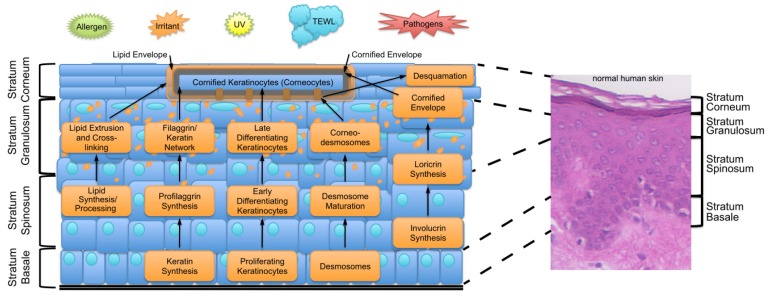
Schematic representation of the underlying differentiation process in keratinocytes. Keratinocytes differentiate from a proliferating state in the basal layer (*Stratum basale*) to dead corneocytes in the outermost layer (*Stratum corneum*). During this differentiation process the lipid envelope, the filaggrin/keratin network, and the cornified envelope is formed and desmosomes mature to corneodesmosomes. Together these components form a compact barrier against the outside preventing entry of harmful components, for example allergens, pathogens, irradiation and other irritants, into the skin and body. Furthermore the barrier inhibits the trans-epidermal water loss (TEWL) and associated loss of solutes. Dotted lines indicate where the sections in the scheme are localized in the normal human skin.

The epidermis is the outermost compartment of the skin and provides a barrier against the outside. This barrier can be divided into three lines of defense: the physical barrier against pathogens and mechanical injuries, the chemical/biochemical barrier with antimicrobial activity, and a barrier against the unregulated loss of water and solutes. The skin barrier is formed by differentiating keratinocytes and continuously renewed. During the differentiation (also called cornification, which refers to the final differentiation steps) keratinocytes undergo different developmental stages from cycling keratinocytes in the stratum basale (SB), through early to late differentiating cells in the stratum spinosum (SS) and the stratum granulosum (SG) to dead cornified corneocytes in the stratum corneum (SC). This cornification is a highly complex process involving many structural proteins, fatty acids and lipids, and processing enzymes. Their function and expression is under the control of cytokines and intercellular signaling molecules. Thus all components need to be highly regulated in a differentiation-associated manner, *i.e.*, their timely and quantitative expression, to ensure the proper development of the epidermal barrier. In Figure 1 the epidermal barrier and the processes, which are needed to develop an appropriate barrier, are summarized. The genes encoding many of the structural and regulatory proteins are localized in the epidermal differentiation complex (EDC) on human chromosome 1q21 (For review see: [7,8]). During cornification the keratinocytes develop a protein- and lipid-rich peripheral envelope, called the CE. This structure is composed of several proteins, most notably involucrin and loricrin, and linked to the CE through keratin filaments. These are stabilized by crosslinking of filaggrin [9]. In the outermost layer of the CE, ceramides and other lipids are covalently bound and form the so-called lipid envelope. These lipids are synthesized and stored in lamellar granules, which are extruded into the extracellular space in the upper granular layer during cornification. The main function of the lipid envelope is to prevent trans-epidermal water loss (TEWL) and the loss of solutes. In summary the cornified layer is composed of dead, flattened, cornified corneocytes. These cells, tightly linked to each other by modified desmosomal structures (corneodesmosomes), are surrounded by insoluble lipids. Additionally to this compact and mechanically stable structure, keratinocytes synthesize and secrete diverse antimicrobial peptides (AMPs), like psoriasin, calprotectin or Rnase7, and free fatty acids, which also have antimicrobial properties, during the terminal differentiation process [10]. AMPs are interacting with and binding to the lipid envelope and together provide the functions to kill or inactivate microorganisms when they come into contact with the epidermis (for review see: [11]). In addition keratinocytes secrete inflammation-associated AMPs like defensins and cathelicidn LL-37 as a direct inflammatory response upon barrier disruption and pathogen invasion [10]. The continuous renewing process is balanced by shedding of the “old” corneocytes from the surface of the epidermis. This process, which is referred to as desquamation, is not simply a mechanical loss of cells. Desquamation is tightly controlled by specific proteases that weaken the cohesion of the SC by degrading e.g., corneodesmosomal proteins under specific conditions that are achieved in the upper layers of the epidermis. A main trigger is the increasing concentration of Ca^2+^ from the basal cell layer to the SC as many proteases and protein modifying enzymes involved in differentiation and desquamation are dependent on defined Ca^2+^ concentrations. This process is essential for the periodical renewal of the skin barrier and defects in desquamation can result in disturbed epidermal barrier (for a more detailed discussion of the skin barrier see [1,12]). Alterations and disorganization of the differentiation process, including modified lipid composition, lead to weakening of the skin barrier, allowing entry of environmental allergens, pathogens and harmful agents into the skin. To maintain the function of the epidermal barrier many processes have to be synchronized and many different proteins have to be expressed and processed at particular stages of differentiation. Many cytokines are able to influence these processes, including cornification. The functions of cytokines in healthy skin but also the consequences of their presence or absence in inflammatory skin diseases are highlighted in this review.

## 3. Cytokine Families

**Figure 2:**
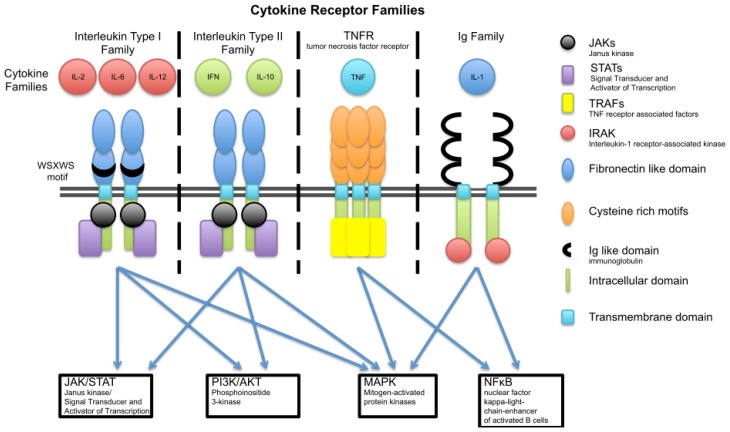
Cytokine receptor complexes, their ligand families and corresponding signaling pathways involved in skin barrier regulation. The cytokine receptor families are grouped according to the three-dimensional structure of the receptors. The receptors of the interleukin type I and interleukin type II families possess extracellular fibronectin like domains. These two families differ only in the WSXWS motif present in the type I, but not type II, receptors. The tumor necrosis factor receptor (TNFR) family has cysteine-rich motifs in their extracellular regions able to bind ligands. The immunoglobulin (Ig) superfamily shares extracellular regions structural homology with immunoglobulin domains.

Cytokines and their receptors can be classified based on the three-dimensional structure of the receptors. This classification results in six groups: (1) Interleukin type I cytokine receptors with a conserved WSXWS amino acid motif in the extracellular domain; (2) Interleukin type II cytokine receptors are similar to type I receptors but lack this conserved motif; (3) The Immunoglobulin (Ig) superfamily shares in their extracellular part structural homology with immunoglobulin domains; (4) The tumor necrosis factor receptor (TNFR) family shares a cysteine-rich extracellular cytokine-binding domain; (5) The IL-17 family of cytokines shares only little homology with the receptors of the other cytokine families. Finally in group 6 are chemokine receptors that couple to G proteins. In Figure 2 the cytokine receptor families mentioned in this review are summarized together with their signaling pathways. In this review we will exclusively concentrate on cytokines belonging to the above mentioned cytokine families that are reported to have an impact on skin barrier function.

### 3.1. Interleukin Type I

The interleukin type I cytokine receptors are subdivided into five sub-groups. The cytokines mentioned in this review can be divided into the following sub-groups. The γ-chain containing IL-2 family receptors for the cytokines IL-4, IL-13 and IL-21 [13]; the IL-6 or gp130 family, a sub-group that includes gp130 in their receptor complexes with the members IL-6, IL-31 and OSM [14]; the IL-12 family consisting of the receptors for IL-23 [15]. The extracellular part of the interleukin type I receptor family consist of fibronectin like domains with a WSXWS domain specific for this type of receptor family. Signaling of these receptors through their intracellular parts activates the JAK/STAT, MAP kinase and PI3K/AKT pathways.

#### 3.1.1. γ-Chain (IL-2) Family

In the following we briefly discuss those IL-2 family cytokines that are relevant for the skin barrier. These include IL-4, the closely related cytokine IL-13 and IL-21.

##### 3.1.1.1. IL-4 and IL-13

IL-4 and IL-13 induce immunoglobulin E (IgE) production *in vitro*[11,16], and their mRNA levels are strongly up-regulated in biopsy specimens of allergic contact dermatitis as well as atopic dermatitis patients [17]. Therefore the role of IL-4 and IL-13 in the pathogenesis of AD was extensively studied. *IL4* is predominantly expressed in the initial phase of acute skin inflammation, in contrast to the later chronic phase of AD [18]. Treatment of calcium-induced differentiating keratinocytes with IL-4 in combination with IL-13 leads to a reduction of *FLG* (filaggrin), *LOR* (loricrin) and *IVL* (involucrin) gene expression [19–22]. Treatment with IL-4 and IL-13 alone reduces the expression of *FLG2*, and *HRNR* (hornerin) that are both structurally related to filaggrin and involved in the epidermal barrier formation [23] These genes are localized on chromosome 1q21 in the EDC [24] and are essential for the maturation of the human epidermis [25]. Loricrin and involucrin are the major precursor proteins for the CE and an altered expression of these proteins results in barrier dysfunction [8,26]. Treatment of keratinocytes with IL-4, IL-13 or both also result in a significant down-regulation of caspase-14 synthesis [27]. Caspase 14 is a protease required for the processing of filaggrin to natural moisturizing factors [28]. Caspase-14 activation correlates with the induction of cornification, pointing to its role in the terminal differentiation process of keratinocytes [29]. Furthermore, IL-4 and IL-13 treatment significantly induces the release of the peptidase KLK7 from human keratinocytes, which is directly involved in the degradation of corneodesmosomal proteins such as desmoglein 1, desmocollin 1, and corneodesmosin to initiate skin desquamation. IL-4 treatment of keratinocytes decreases the amount of corneodesmosome formation and down-regulates the expression of desmoglein 1 [30]. Repression of CE structural protein expression and enhanced KLK7 expression results in enhanced skin desquamation [12,31] and increased TEWL [30,32]. In contrast to the enhanced expression of KLK7, expression levels of the serine proteases KLK5 and KLK14 are decreased after IL-4 treatment [30]. IL-4 treatment also negatively influences the ceramide synthesis in the SC, inhibits the gene and protein expression of the corresponding metabolic enzymes and alters their enzymatic activities as summarized in [33]. These results are consistent with observations made in transgenic mice that overexpress IL-4 ubiquitously under the control of the MHC class I regulatory sequence. They develop acanthosis (epidermal hyperplasia, implies increased thickness of the SB and SS), hyperkeratosis and dermal collagen deposition, as well as mast cell accumulation in the skin. Mice treated with recombinant IL-4 show a reduced SC thickness and a reduced cohesion of the SC measured by the amount of protein removed from the skin by repeated tape stripping [30]. Keratinocytes isolated from these mice are hyperproliferative [34]. Moreover a transgenic mouse line expressing IL-4 under the control of the keratin 14 promoter in the epidermis, spontaneously develop pruritic inflammatory skin lesions [35]. Consistent with the phenotypes of the above-described transgenic mice, IL-4^−/−^ mice develop a strengthened skin barrier with increased filaggrin and involucrin protein expression [36], indicating that IL-4 has the ability to regulate the expression of EDC genes and may play an important role in the physiological regulation of epidermal homeostasis and innate barrier function.

IL-13 transgenic mice develop inflammatory skin lesions on the back and abdomen with hair loss, dry skin, excoriation, crusting, and bacterial pyoderma. IL-13 induces fibrosis and increased vasculature in transgenic mice [37]. IL-13 has two cognate receptors, the IL-13Rα1 and the IL-13Rα2. Deletion of the IL-13Rα2 results in an aggravated IL-13 response because the IL-13Rα2 receptor acts as a decoy receptor that inhibits the activity of its ligand. Mice lacking the IL-13Rα2 receptor have an enhanced response to IL-13 leading to defects in skin barrier formation and development of cutaneous inflammations. In the absence of any stimulus no inflammatory phenotype is observed in IL-13Rα2^−/−^ mice, however following *Aspergillus fumigatus* infection enhanced TEWL and inflammation, increased peripheral eosinophilia and IgE levels were measured compared to wild-type animals. *Aspergillus fumigatus* serves as a model for epicutaneous allergen-sensitization and allergen challenging. From these experiments it was concluded that IL-13Rα2 plays a role in the epidermal barrier function by negative regulating the IL-13 signaling [38].

These results provide evidence for a role of IL-4 and IL-13 in the barrier function of the skin. Overexpression of IL-4 and IL-13 in the skin seems not to be sufficient for the induction of a fully developed atopic dermatitis phenotype. Nevertheless both cytokines are able to generate an inflammatory skin phenotype and thus weaken the skin barrier. These results are confirmed by the fact that mice lacking the transcription factor STAT6, which is the major downstream signal transducer in the pathway of both IL-4 and IL-13, can develop AD like lesions [39]. Both cytokines signal through the IL-4Rα receptor, which is a component of two receptor systems. The type I receptor is composed of IL-4Rα and γ-chain and the type II receptor of IL-4Rα and IL-13Rα1. Il-4 is able to bind to the IL-4Rα chain independent of the second receptor subunit, whereas IL-13 is only able to bind the IL-13Rα1 subunit in this heterodimeric receptor. Because both cytokines signal via different receptor complexes they are also able to induce different signal transductions pathways. The γ-chain is able to induce the PI3K/AKT pathway as well as the MEK1/2, ERK1/2 pathway [40]. The IL-13Rα1 subunit is not able to induce this pathways, this subunit activates Tyk2 and JAK2. The effect on the skin barrier might also be a cooperative effect of these pathways induced by the individual subunits. This indicates that neither IL-4 nor IL-13 alone might be required for the disease establishment, but they seem to be important mediators in regulating, either directly and/or indirectly, skin barrier formation associated genes and, thereby exert control on skin homeostasis and innate barrier function.

##### 3.1.1.2. IL-21

IL-21 and the IL-21 receptor expression are increased in skin lesions of human AD patients, whereas IL-21 is not detectable in normal human skin. *IL21* and *IL21R* are also up-regulated following mechanical skin injury like tape stripping. Mice lacking the IL-21 receptor show impaired allergic skin inflammation and systemic response to sensitization, which appears to be the result of a defective migration of antigen-presenting dendritic cells (DCs) [41]. Intradermal injection of IL-21 into mice skin causes an increased proliferation rate of keratinocytes and in consequence epidermal hyperplasia. In a human psoriasis xenograft mouse model the blockade of IL-21 activity by anti-IL-21 antibodies resolves IL-21-induced inflammation and reduces keratinocyte proliferation [42]. Studies in knockout mice demonstrate that IFN-γ is necessary for IL-21-induced epidermal hyperplasia, suggesting that IFN-γ is a downstream effector of IL-21 [43]. These data indicate that this cytokine influences the formation and quality of the skin barrier indirectly by modulating the inflammatory response.

Increased expression of IL-21 in lesions of AD patients results most likely in the invasion and activation of CD4^+^ T-cells, natural killer T-cells, and follicular T-cells [44,45]. Scratching and other mechanical injuries could further enhance the migration of DCs triggered by IL-21 and could so increase the disease severity by a more efficient sensitization to environmental allergens.

#### 3.1.2. The IL-6 Family of Cytokines

In the following we briefly discuss those IL-6 family cytokines that are relevant to the skin. These include IL-6 itself and additionally IL-31 and OSM.

##### 3.1.2.1. IL-6

The levels of IL-6 in serum and in skin blister fluids correlate with the severity of psoriasis [46]. IL-6 and IL-6 receptor expression is increased and localized to all nucleated epidermal layers after barrier disruption. In a human epidermal keratinization model IL-6 reduces the amount of ceramide in the SC but increases the total epidermal ceramide level. The expression of genes encoding key enzymes accountable for the synthesis of ceramides was also inhibited by IL-6 treatment [33]. IL-6 is able to increase keratinocyte proliferation and epidermal thickening in an transgenic mouse model [47,48]. In agreement with these results, IL6^−/−^ mice exhibit a delay in skin permeability barrier repair after tape stripping whereas the application of IL-6 enhances this repair process in a concentration dependent manner [49]. This is at least in part explained by the finding that IL-6 promotes the expression of the vascular endothelial growth factor (VEGF) [50]. VEGF gene expression levels were reduced in IL-6^−/−^ mice at wound sites compared to control mice, and also wound healing was delayed, with delayed angiogenesis and collagen deposition [51].

All these results suggest a role for IL-6 in wound healing by promoting keratinocyte proliferation, angiogenesis via VEGF and collagen deposition. Increased IL-6 levels in psoriasis patients could be due to a significant disruption of the skin barrier and the associated enhanced risk of pathogen invasion that promotes the synthesis of IL-6 by immune cells. However a direct role of IL-6 in the formation of the skin barrier has not been observed yet. Therapeutically, IL-6 might be useful to treat skin with a disrupted barrier to enhance the re-epithelization and promote wound healing.

##### 3.1.2.2. IL-31 and OSM

AD patients show significant higher levels of IL-31 in serum and increased *IL31* mRNA levels in lesional skin samples [21]. These higher levels are correlated with serum IgE, disease severity, and subjective itch intensity [5,17,21,52,53]. IL-31 signals via its heterodimeric receptor complex composed of the IL-31RA and the OSMRβ [54]. IL-31 interferes with keratinocyte differentiation in organotypic 3D cell culture models, where the keratinocytes at the air-liquid interphase are able to differentiate into a skin-like structure. Treatment with IL-31 leads to a reduced epidermal thickness, disturbed epidermal constitution, altered alignment of the SB and poor development of the SG. The observed differentiation defect is associated with a profound repression of terminal differentiation markers, including filaggrin, an essential factor for skin barrier formation, and a reduced lipid envelope [55]. Transgenic mice overexpressing IL-31 develop severe pruritus, alopecia (hair loss) and skin lesions [54]. IL-31 may also play a role in the sensation of itch, because several itch-associated skin diseases like prurigo nodularis [56], chronic spontaneous urticaria [57], and allergic contact dermatitis [17] exhibit high levels of IL-31. Treatment of Nc/Nga mice (a model for AD) [58,59] with anti-IL-31 antibodies leads to a reduction in scratching behavior. Dorsal root ganglia that are meant to be responsible for the sensation of itch express the IL-31 receptor and their unmyelinated C fibers might be directly stimulated by IL-31. It might also be possible that IL-31 triggers the expression of pruritic factors, which subsequently activate the C fibers leading to the sensation of itch [60]. As this is mediated by neurons, the regulation of pruritus by IL-31 might be completely unrelated to its repression of keratinocyte differentiation.

Both OSM production and OSM receptor expression are enhanced in inflammatory skin diseases. OSM is a potent inducer of keratinocyte migration and triggers hyperplasia of reconstituted human epidermis [61]. OSM suppresses the expression of the “classical” epidermal differentiation markers (e.g., filaggrin) and leads to a reduction of cornified cells in reconstituted human epidermis [62]. Cell and skin models show similar phenotypes when they are treated with either IL-31 or OSM. This could be due to the similarity of both receptor complexes. So far IL-31 is the only cytokine of the IL-6 family that does not signal through a receptor complex that contains gp130, which makes it to a unique member within this family. Instead it signals through a heterodimeric receptor complex composed of the IL-31RA, which is related to gp130, and the OSMRβ [60]. Thus to understand IL-31 signaling the expression pattern of the OSMRβ needs to be considered.

High serum levels of IL-31 in AD patients, impaired differentiation in organotypic models (that do not include inflammatory cells or factors besides the ones that may be produced by keratinocytes), and the increased scratching behavior in AD mice provoked the conclusion that high IL-31 expression is one of the causes rather than only a symptom of AD. This suggests that anti-IL-31 treatment, either by blocking the IL-31 receptor complex or by reducing IL-31 cytokine levels, may be beneficial for patients.

#### 3.1.3. IL-12 Family

Relatively little is known about the molecular mechanism of IL-12 family members, *i.e.*, IL-12, IL-23, IL-27, and IL-35, in the regulation of skin differentiation and associated diseases. Elevated levels of *IL23* mRNA are associated with human psoriasis. IL-23 induces epidermal thickening in mice due to keratinocyte hyperplasia in the spinous layer and altered granular layer differentiation. In contrast in *in vitro* cell proliferation assays IL-23 has no effect on keratinocyte proliferation [63]. IL-23 treatment of mice enhances gene expression of *IL19* and *IL24*[63]. The injection of either IL-12 or IL-23 into the skin of mouse ear stimulates acanthosis and inflammation [64].

This indicated a role of IL-23 in the differentiation process of the skin, which do not affect proliferation in 2D and therefore a role in the pathogenesis of skin disease. It is most likely that in mouse models IL-23 is able to enhance the expression of other cytokines like TNF by macrophages [65] that are known to induce hyperplasia as *in vitro* assays show no effect of IL-23 on keratinocytes. Blocking IL-23 using monoclonal antibodies are approved therapeutic approaches in psoriasis treatment [66]. The function of IL-23 needs to be evaluated more carefully to be able to draw conclusions about its role in skin barrier formation.

### 3.2. Interleukin Type II Cytokine Receptors

Type II cytokine receptors are divided into three sub-groups, *i.e.*, the IL-10 family and the type I and type II interferon families. All three sub-groups have been found to affect the proliferation and differentiation of keratinocytes. In particular the IL-10 family with IL-19, IL-20, IL-22 and IL-24 [67] has various effects on the skin. Like the Interleukin type I cytokine receptors the type II receptors use the same signaling pathways (MAP kinase, JAK/STAT, and PI3K/AKT).

#### 3.2.1. IL-10 Family

##### 3.2.1.1. IL-19

The gene expression of *IL19* is increased in inflammatory skin diseases [68] and in particular in lesional psoriatic skin compared to non-lesional psoriatic skin [69]. Treatment of a human reconstituted epidermis with IL-19 induces hyperplasia of the cells in the lower layers of the epidermis, *i.e.*, the basal and the spinous layers, while it has little or no apparent effect on either the granular cell layer or the SC [70]. Mice transgenic for IL-19, have no overt skin phenotype [71]. These findings indicate that IL-19 might only play a minor role regulating the formation of the skin barrier.

##### 3.2.1.2. IL-20

Like *IL19*, the expression of the *IL20* gene is also increased in inflammatory skin diseases like AD or psoriasis [68] and *IL20* mRNA is upregulated in lesional compared to non-lesional psoriatic skin [69]. IL-20 induces proliferation of keratinocytes in monolayer culture, even if a neutralizing antibody blocks EGFR-mediated autocrine growth. In line with these results, IL-20 induces acanthosis or hyperplasia in stratified epidermal culture systems and epidermal thickening in the early cell layers in a dose dependent manner [70]. In organotypic 3D co-cultures with fibroblasts IL-31 is able to induce the expression of *IL20* and *IL24*, suggesting that these two cytokines are downstream effectors of IL-31. Indeed, IL-20 and IL-24 possess a small inhibitory effect on the differentiation of HaCaT cells in the 3D model. This phenotype is also characterized by a reduced expression of filaggrin and keratin 10 [55]. It is worth mentioning that these effects are relatively minor compared to IL-31, indicating that IL-31 induces additional mediators that control keratinocyte differentiation.

Overexpression of IL-20 in transgenic mice causes neonatal lethality with skin abnormalities including aberrant epidermal expression of several keratins indicating a much more prominent role of IL-20 in epidermal development during embryogenesis [72]. Histological analyses of the skin of different IL-20 transgenic mice display a thickened epidermis, hyperkeratosis, a compact SC and they express differentiation and proliferation markers that are normally confined to the basal and suprabasal layers of the skin. Overexpressing IL-20 in other tissues also causes these changes, indicating that circulating and epidermis infiltrating IL-20 is most likely responsible for the observed phenotype [73].

##### 3.2.1.3. IL-22

*IL22* gene expression is elevated in inflammatory skin diseases like psoriasis or AD [74]. IL-22 induces keratinocyte migration *in vitro*, triggers hyperplasia of reconstituted human epidermis [75] and down-regulates filaggrin, cathepsin D and calpain expression [76]. Cathepsin D is an aspartate protease, which increases the activity of transglutaminase 1, which cross-links the cornified envelope proteins involucrin and loricrin during epidermal differentiation [77]. Calpains are intracellular calcium-dependent cystein proteases that are involved in the processing of filaggrin during terminal differentiation of keratinocytes [78]. IL-22 inhibits the mRNA expression of the genes encoding kallikrein 7 (KLK7), desmocollin 1 (DSC1) and calmodulin-like 5 (CALML5) in keratinocytes and induces the expression of MMP3 [79]. DSC1 is an adhesive desmosomal protein and the degradation of these proteins at the epidermal surface by KLK7 is necessary for the physiological desquamation of stratified epithelia [79]. CALML5 is a calcium binding protein that interacts with transglutaminase 3, another key cross-linking enzyme during the terminal differentiation of keratinocytes [80]. IL-22 inhibits the expression of the late cornified envelope protein 1B [81], which is also located within the EDC on chromosome 1q21[24]. These observations suggest that IL-22 influences the epidermal barrier by affecting proteins that are involved in the processing of proteins relevant for the barrier, *i.e.*, most likely proteins that are involved in filaggrin processing and desquamation. In addition to these effects, IL-22 induces proliferation of keratinocytes in monolayer cultures and, in line with these experiments, IL-22 induces acanthosis and hyperplasia in stratified epidermal culture systems and epidermal thickening of the early cell layers in a dose dependent manner [82]. IL-22 also enhances cell migration and triggers hyperplasia in a reconstituted human epidermal models but no change in proliferation was observed. This was associated with reduced expression of involucrin, loricrin, keratin 10, and filaggrin [75]. Together these studies document that IL-22 controls many key components of the SC and thus appears to have the capacity to profoundly affect skin differentiation.

The treatment with IL-22-neutralizing antibodies in a psoriasis-like mouse model prevents the development of symptoms, in particular acanthosis is ameliorated and inflammatory infiltrates and the expression of Th17 cytokines is reduced. In line with these findings the direct administration of IL-22 into the skin of normal mice induces the expression of genes that encode antimicrobial peptides and proinflammatory cytokines [83]. Moreover IL-22 induces *MMP1* mRNA expression [79]. In contrast to the application of IL-22, IL-22 transgenic mice exhibit a different psoriasis-like phenotype. The granular layer of transgenic animals contains fewer keratohyalin granules and the cornified layer is more compact than that of wildtype mice. Importantly, the skin alterations of IL-22 transgenic mice recapitulate the main features of psoriatic skin [84], further documenting the decisive role of this cytokine.

In summary these date suggest a role for IL-22 in the pathogenesis of psoriasis. IL-22 treatment leads to increased proliferation and less differentiation of keratinocytes. IL-22 inhibits either directly via abrogating the expression of differentiation marker genes or indirectly via inhibiting important enzymes the correct cross-linking of the epidermal layer and a proper desquamation. Furthermore IL-22 is able to induce a psoriasis-like phenotype in mice and blocking IL-22 by applying anti-IL-22 antibodies abates disease symptoms in this model. Thus IL-22 is a potential target in the therapy of psoriasis.

##### 3.2.1.4. IL-24

IL-24 expression is increased in inflammatory skin disease like AD or psoriasis [68] and IL-24 induces, as well as the other IL-10 family members IL-19 and IL-20, the proliferation of keratinocytes in 2D cultures. Like IL-19 and IL-20, IL-24 induces acanthosis/hyperplasia in a reconstituted human epidermis culture system and epidermal thickening in the early cell layers in a dose dependent manner. IL-24 decreases *KRT10* mRNA expression [75] while it increases *KRT16*[70]. The latter is upregulated in the suprabasal layers of interfollicular epidermis showing hyperproliferation and abnormal differentiation and thus also serves as a psoriasis marker protein [85]. IL-24 is able to inhibit TGFα-induced migration of keratinocytes in an *in vitro* model of wound repair and interferes with TGFα-mediated proliferation of keratinocytes[86]. In organotypic co-cultures with fibroblasts, IL-24 together with IL-20 impairs differentiation and reduces the expression of *FIL* and *KRT10*[55]. In transgenic mice IL-24 leads to an increase of epidermal thickness [87]. Together these findings document that IL-24 function is distinct in normal skin compared to skin with an inflammatory response. In the former, IL-24 increases the proliferation and impairs normal differentiation whereas in inflamed skin proliferation and migration of keratinocytes is inhibited. This suggests that the consequences of IL-24 are modulated or negated by other cytokines/signals present during inflammation. It will be interesting to define the nature of these factors that crosstalk with IL-24 signaling.

These observations indicate a number of different roles for the cytokines of the IL-10 family (with the exception of IL-10 itself, whose function is rather distinct in comparison to the other family members) in regulating keratinocyte proliferation and differentiation in the epidermal layer. *IL19*, *IL20* and *IL24* are clustered together with *IL10* in the *IL10* gene cluster on chromosome 1q32 whereas *IL22* is located on chromosome 12q14 [72,88]. This clustering of the genes and the fact that IL-19, IL-20 and IL-24 signal via the same receptor components (IL-20R1, IL-20R2, IL-22R1) and thus are able to activate the same signaling pathways may be one reason for these overlapping but not identical phenotypes in response to these cytokines. It seems that all these cytokines have a role in regulating the skin barrier by controlling keratinocyte proliferation and migration as well as abrogating the expression of genes that encode components of the skin barrier.

#### 3.2.2. Interferons

The family of interferons can be subdivided into two groups of interferons. Group I interferons, which binds to IFNA receptors (IFNAR), include IFNα, IFNβ and IFNω. IFNγ is the only member of the group II interferons, which interacts with the INFG receptors (IFNGR).

##### IFNα and IFNγ

*IFNG* expression is increased in inflammatory skin disease like AD or psoriasis [68,74]. In these diseases higher levels of IFNα and γ can alter the response to different cytokines by regulating the expression of their corresponding receptors. It was shown that IFNγ enhances the expression of the IL-31 receptor [89,90] and of the OSM receptor [91], while reducing IL-4 receptor expression [92], whereas IFNα enhances the expression of the IL-22 receptor [93]. IFNα as well as IFNγ have a modulatory effect on the expression of the intergrins α3, α6 and β1. IFNα treatment has an inhibitory effect on integrin α6 expression in basal cells of reconstructed skin [94]. The integrin α6 and β4 are main components of the hemidesmosomes, which are essential protein complexes that mediate the firm attachment of epithelial cells to the underlying basement membranes [95]. Mice with a conditional ablation of integrin α6 in the epidermis results in skin fragility and inflammation [96], the latter probably as a consequence of impaired barrier formation. Moreover α3β1 integrins play crucial roles in the organization of epithelial and endothelial tissues by exerting functions related to cell adhesion and migration [97]. In contrast to the decreased epidermis-dermis interaction, the levels of transcripts for acid sphingomyelinase (SMase) and glucocerebrosidase (GCase), both enzymes play important roles in ceramide synthesis, are stimulated in response to IFNγ[33]. SMase hydrolyses sphingomyelin thereby producing ceramides 2 and 5 [98], whereas GCase degrades glucosylceramide to produce the ceramides 1 to 7 [99]. Ceramides are an integral part of the lipid envelope accounting for 30%–40% of stratum corneum lipids by weight. In combination with cholesterol and free fatty acids (FA), ceramides form the extracellular lamellar membrane structures. This subserves the permeability barrier and is important to prevent water loss due to evaporation [12]. Thus through activation of the expression of SMase and GCase, INFγ enhances ceramide synthesis and consequently strengthens the lipid envelope. Consistent with these findings is that IFNγ suppresses TEWL [32].

In conclusion, IFNs seem to play a role in maintaining the barrier function of the skin by regulating receptors important for cytokine signaling, including the IL-4 and IL-31 receptors. Cytokines belonging to these receptors have a tremendous impact on the formation of the skin barrier (see above). Furthermore IFNs seem to directly influence the detachment of keratinocytes from the basal membrane, an essential step in the differentiation process from basal to primary differentiating suprabasal cells. Furthermore IFNs are involved in ceramide synthesis and therefore influence the formation of the lipid envelope and thus are able to regulate TEWL.

### 3.3. Ig Superfamily (Receptors with Extracellular Immunoglobulin (Ig)-Like Domains)

The IL-1 family of cytokines includes a growing number of members that play important roles in immune regulation and inflammatory processes (summarized in [100]). Here we address the functions of those family members, *i.e.*, IL-1α and IL-1β, that have been analyzed in combination with epidermal differentiation and skin diseases.

#### IL-1α and IL-1β

The cutaneous expression of *IL1A* transcripts is reduced in inflammatory skin diseases like psoriasis or AD whereas no significant changes are seen for *IL1B*[74]. However an increase of secreted IL-1α in lesional skin of psoriasis patients was observed [101]. After acute permeability barrier disruption, an increase in the epidermal expression of IL-1α and IL-1β occurs, which might have protective function [102]. Indeed stimulating keratinocytes with IL-1α enhances the synthesis of barrier lipids in monolayer cell culture models [103]. In contrast O’Shaughnessy and colleagues found that IL-1α leads to hyperkeratosis without increased lipid synthesis and causes a reduction of keratin 10 and involucrin expression. Moreover IL-1α promotes the expression of the fatty acid desaturase 2 in organotypic 3D cultures [104]. These contrasting findings regarding the influence of IL-1α on lipid synthesis in particular could be due to differences in the experimental setup. Barland and colleagues used calcium differentiated keratinocytes in a monolayer culture [103], while O’Shaughnessy *et al.* treated organotypic 3D cultures with IL-1α [104]. 3D co-cultures with fibroblasts serve as a more physiological model for epidermal development and homeostasis, in which the Ca^2+^ gradient develops in the epidermal layer comparably to normal skin. Moreover the fibroblasts provide physiologically relevant cytokines and growth factors. In contrast Ca^2+^-induced differentiation of a monolayer culture results in a highly simplified version of the differentiation program. This might explain the differences observed in the two models in response to IL-1α. Although this seems an obvious explanation, it should be noted that explicit studies addressing the differences of keratinocyte differentiation between the 3D co-culture model and a monolayer culture treated with Ca^2+^ are missing. In other words it remains open to what extent these different models are comparable. Nevertheless it will be important to define the role of IL-1α in more detail, as this important cytokine is likely to be involved in keratinocyte differentiation.

The intra-cutaneous administration of IL-1α was able to improve barrier function in mice by enhancing the lipid synthesis and the lamellar body formation [103]. In another study, IL-1α injected into mice led to an increased barrier recovery after perturbation and IL-1α stimulation restored epidermal permeability and the antimicrobial barrier that were compromised by topical application of tacrolimus, an immunosuppressive drug [105]. IL-1α up-regulates the expression of genes associated with cell adhesion, proliferation, and epidermal differentiation [106]. Conversely, transgenic mice that overexpress IL-1α in basal keratinocytes under the control of the K14 promoter, develop spontaneous inflammatory skin lesions, as well as dermal neutrophil infiltration even in non-lesional skin [107]. In all epithelial cells the IL-1α precursor is constitutively expressed translocates to the nucleus and can modulate gene transcription and thus modifies cell behavior and cellular differentiation [108]. Overexpressing this biological active precursor in keratinocytes has distinct consequences to the skin barrier in comparison to injecting a biologically active IL-1α that binds to its cell surface receptor and induces intracellular signaling. IL-1α signaling attracts lymphocytes and neutrophils via the expression of CCL20 in keratinocytes [106]. Mice with a knockout of the IL-1 receptor type 1 develop a more profound barrier deficit than age-matched wild-types and an increase in IL-1α protein expression in the skin after acute permeability barrier perturbation. IL-1β treatment leads to an increased expression of the main thigh junction proteins occluding and claudin-1 at early time points after treatment. Furthermore ZO-1, which connects tight junctions with the cytoskeleton, was also up-regulated in organotypic 3D models at early time points after treatment. In contrast to this early response to the IL-1β treatment, after 96 h of stimulation the expression of all these proteins is decreased. In line with the altered expression of these structural proteins, the treatment with IL-1β leads to an increased transepithelial resistance of calcium differentiated keratinocytes as a consequence of the early response and a decreased resistance at later stages, measured with an epithelial voltohmmeter [109].

It appears that IL-1α and IL-1β have profound effects on epidermal differentiation that need to be tightly controlled under physiological conditions. They are able to strengthen the epidermal barrier via influencing the mechanical attachment of cells and the formation of the LE. This might be highly relevant in a response to e.g., mechanical injury of the skin and wound repair. Nevertheless a deregulated expression of IL-1α seems to promote an inflammatory skin phenotype e.g., by attraction of inflammatory cells. Together it seems worth proposing that the role of IL-1α and β in skin physiology and pathology needs to be studied in greater detail, for example to answer the question whether deregulated IL-1 expression occurs in skin diseases and if this might contribute to disease symptoms like barrier disruption.

### 3.4. The TNF Family

Nineteen ligands have been identified as part of the TNF family [110]. Almost all of the TNF proteins are expressed by cells of the immune system, including B cells, T cells, NK cells, dendritic cells, and monocytes [111]. Besides the signaling via the MAP kinase and the NFκB pathways, TNF receptors are able to induce apoptosis after ligand binding.

#### TNFα

TNF serum levels and in suction skin blister fluids correlate with the severity of psoriasis [46]. After acute permeability barrier disruption an increase in the epidermal expression of *TNFA* can be measured [102]. TNFα inhibits *FLG* and *LOR* mRNA expression in calcium differentiated keratinocytes [112]. Levels of transcripts encoding for SMase and GCase and the amount of ceramide in human epidermal sheets are enhanced in response to TNFα [33]. Consistent with this the TEWL is suppressed by TNFα treatment [32]. Application of TNFα after experimental injury of murine skin enhances permeability barrier repair and mice lacking the TNF receptor p55 have a delayed permeability barrier repair [113].

Together, these results suggest that TNFα signaling pathways are important for permeability barrier repair through SMase- and GCase-mediated generation of ceramides. However in situations with pathological levels of TNFα, this cytokine prevents proper barrier formation by inhibiting the expression of *FLG* and *LOR*, resulting in weakening of the skin barrier. Targeting TNFα in psoriasis patients by therapeutics significantly abates disease symptoms [114]. The impact of TNFα signaling and the role of TNF-induced NFκB activation on epidermal physiology is reviewed in [115].

### 3.5. IL-17 Family

The IL-17 cytokine family consist of six members, IL-17A to IL-17F (IL-17A is called IL-17 and IL17E is also called IL-25) [116].These family members are critical players in immunity and inflammation (summarized in [117]). Here we sum up the current knowledge about the family members IL-17 and IL-25 regarding skin barrier.

#### 3.5.1. IL-17

IL-17 is expressed in skin lesions of AD patients and the number of IL-17 producing CD4^+^ T-helper cells (Th-17 cells) cells infiltrating the dermis of atopic eczema patients correlate with the disease severity and significantly more IL-17 positive cells are found in acute compared to chronic lesions [118].

Mechanistically, IL-17 down-regulates the expression of *FLG* as well as the genes that encode filaggrin processing enzymes in cultured keratinocytes. It also inhibits the expression of proteins, such as ZO-1, ZO-2, which are components of tight junctions and desmosomes, and the epidermis-associated adhesion molecule E-cadherin and various integrins [119]. Human organotypic 3D models treated with IL-17 have a decreased epidermal thickness compared to the non-treated control but no change in *KRT10* expressions could be observed [84]. Treatment of psoriasis patients with antibodies targeting IL-17 improved the clinical symptoms of psoriasis [120,121].

#### 3.5.2. IL-25 (IL-17E)

Little is known about the role of IL-25 in skin differentiation. In patients with contact dermatitis and psoriasis, increased levels of IL-25 are present in involved skin in comparison to uninvolved tissue. Moreover in monolayer cultures treated with IL-25 a reduced profilaggrin expression has been documented [122], indicating that this cytokine may indeed play a role in the differentiation and function of the epidermis. Furthermore IL-25 treatment of Ca^2+^ differentiating keratinocytes leads to a decreased expression of filaggrin, hornerin, and filaggrin 2 [23]. This warrants further studies on the role of IL-25 in the skin.

## 4. Concluding Remarks

**Figure 3:**
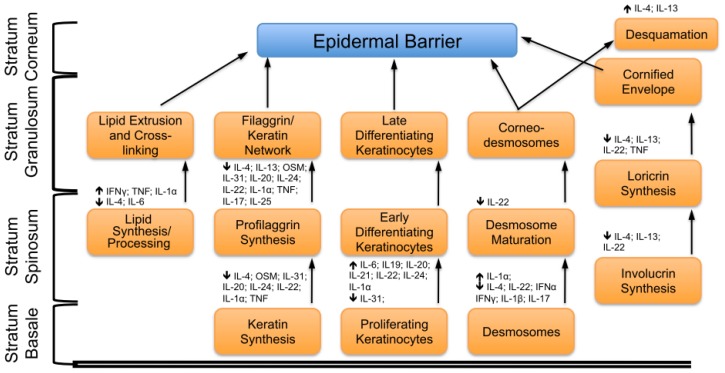
Regulatory effects of the cytokines on the barrier formation. This figure illustrates the relevant effects of cytokines on the formation of the skin barrier and their selected targets in the differentiation process documented either in cell culture or in animal studies. Arrows indicate the consequences on these processes. (↑) indicate that the cytokine enhances or promotes the target process; (↓) indicate that the cytokine down-regulates or inhibits the target process.

In this review we summarize the function of cytokines in skin barrier formation and their potential role in skin disease pathology. The various cytokines act at multiple levels to control barrier formation, like the cornification process (e.g., IL-31), the composition of the lipid envelope (e.g., IFN-γ) and cell–cell adhesion (e.g., IL-1α). All cytokines and their targets in the formation of the skin barrier are summarized in Figure 3. Many cytokines discussed in this review alter the expression of structurally important proteins involved in the formation of the cornified envelope, including loricrin and profilaggrin. Some also prevent the development or maintenance of tight junctions, corneodesmosomes and hemidesmosomes, thereby affecting the interaction of terminally differentiated cells in the cornified envelope or the basal cell layer. Moreover cytokines control the expression of genes that encode important enzymes in processing the proteins and lipids associated with terminal differentiation. Because these processes are highly balanced, it is not surprising that many cytokines provoke a weakening of the barrier, albeit that their effects are mediated by different components. These contribute to efficient barrier formation, beginning with effects on early differentiation processes to the disturbance of terminal events that define the barrier proper. In addition to these “bad” cytokines for barrier formation, others cytokines appear to be important for the efficient repair of barrier activity. This indicates that the interaction of different cytokines will need to be addressed to understand how cytokine balance is relevant for the protective function of the skin.

It should be noted that in most cases it is still unclear what the mechanistic base is for these effects. This is at least in part due to the difficulties in analyzing individual steps during the differentiation process, as this only efficiently occurs *in vivo* or in 3D organotypic cultures. Moreover the terminal differentiation events, which result in the cornified envelop, are difficult to study at the cellular level beyond documenting protein expression by, for example, immuno-histochemical analyses, because the cells no longer proliferate and cannot easily be separated from earlier differentiation stages. As a consequence, in most situations it is not clear how a specific cytokine controls the expression of genes relevant for the differentiation process. Together, the studies that are presently available do not allow precise conclusions about how certain cytokines affect barrier formation. In particular, it is typically not known whether a cytokine controls the expression of a relevant gene directly, or whether these are secondary effects.

The mechanistic studies are further complicated in, for example, mouse skin by many additional cells beyond keratinocytes that infiltrate the epidermis. Many of these are immune cells that respond to different cytokines and also produce cytokine cocktails making the analysis of individual cytokines complex. Nevertheless, it is striking that the majority of the cytokines, which disrupt the barrier function of the epidermis in model systems, are also deregulated in skin diseases, including atopic dermatitis and psoriasis. Moreover the overexpression of these cytokines typically correlates with disease severity. An additional important level of complexity is given by the fact that the disruption of the skin barrier facilitates the invasion of pathogens and allergens. In turn this contributes to local inflammatory immune responses. In other words, deregulated cytokine expression in the skin can weaken barrier function and thus aggravate the inflammatory response, thereby establishing a viscious circle. Thus it is quite obvious that the management of cytokine expression and activity is important to control and ameliorate disease development in the skin.
